# Current Management of Pancreatic Neuroendocrine Tumors: From Demolitive Surgery to Observation

**DOI:** 10.1155/2018/9647247

**Published:** 2018-07-22

**Authors:** Ilenia Bartolini, Lapo Bencini, Matteo Risaliti, Maria Novella Ringressi, Luca Moraldi, Antonio Taddei

**Affiliations:** ^1^Department of Surgery and Translational Medicine, AOU Careggi, University of Florence, Largo Brambilla 3, 50134 Florence, Italy; ^2^Department of Oncology, AOU Careggi, Largo Brambilla 3, 50134 Florence, Italy

## Abstract

Incidental diagnosis of pancreatic neuroendocrine tumors (PanNETs) greatly increased in the last years. In particular, more frequent diagnosis of small PanNETs leads to many challenging clinical decisions. These tumors are mostly indolent, although a percentage (up to 39%) may reveal an aggressive behaviour despite the small size. Therefore, there is still no unanimity about the best management of tumor smaller than 2 cm. The risks of under/overtreatment should be carefully evaluated with the patient and balanced with the potential morbidities related to surgery. The importance of the Ki-67 index as a prognostic factor is still debated as well. Whenever technically feasible, parenchyma-sparing surgeries lead to the best chance of organ preservation. Lymphadenectomy seems to be another important prognostic issue and, according to recent findings, should be performed in noninsulinoma patients. In the case of enucleation of the lesion, a lymph nodal sampling should always be considered. The relatively recent introduction of minimally invasive techniques (robotic) is a valuable option to deal with these tumors. The current management of PanNETs is analysed throughout the many available published guidelines and evidences with the aim of helping clinicians in the difficult decision-making process.

## 1. Introduction

In the last decades, the incidental diagnosis of neoplasms has been greatly increased due to the widespread use of advanced imaging techniques. Indeed, the diagnosis of pancreatic neuroendocrine tumors (PanNETs) has increased fourfold to sevenfold [1]. Furthermore, the size of these lesions at diagnosis has considerably decreased [2, 3], and the detection of tumors < 2 cm ranges from 26% to 61% [4, 5].

Pancreatic neuroendocrine tumors comprised less than 5% of all pancreatic tumors and 7% of all NETs [6, 7] being the second most common pancreatic neoplasm, with an overall incidence of approximately 5 : 1,000,000 new cases/year and an estimated prevalence of 1 : 100,000 people [7, 8]. Actually, they probably represent up to 10% of pancreatic tumors [9]. Moreover, their prevalence at autopsy ranges from 0.8% to 10% [10].

The great majority of PanNETs are sporadic (noninherited), while 10–30% of the patients develop a PanNETs within a genetic syndrome. The most frequent syndrome is multiple endocrine neoplasia (MEN) type 1 [11] while other rare genetic conditions are MEN4, Von Hippel-Lindau disease, neurofibromatosis 1 (von Recklinghausen's syndrome), and tuberous sclerosis [11–13].

Up to 90% of PanNETs are classified as nonfunctional (NF-PanNETs). This group includes also patients presenting with high hormone levels without symptoms. However, a considerable part of these patients (up to 60%) have a metastatic disease at diagnosis, while 21% present a locally advanced disease [10, 14]. Those patients who have nonspecific symptoms complain for abdominal pain, weight loss, or mass effect related to the pancreatic tumor or to the distant spread [13].

Functional PanNETs (F-PanNETs) comprehend insulinomas (35–40% of F-PanNETs) manifesting with the classical Whipple's triad (fasting hypoglycemia, symptoms of hypoglycemia, and immediate relief of symptoms after the administration of glucose) [12], gastrinomas (16–30%) with the Zollinger-Ellison syndrome (multiple peptic ulcers, esophageal reflux, and diarrhea), glucagonomas (<10%) with the “4D syndrome” (dermatitis, diabetes, deep vein thrombosis, and depression), and VIPomas (<10%) related to the Verner-Morrison syndrome (watery diarrhea, achlorhydria, and hypokalemia). The remaining 5% are somatostatinomas, related to combined symptoms such as diabetes, diarrhea, steatorrhea, anemia, and weight loss [11, 15].

From a curative perspective, all patients presenting with F-PanNETs should be evaluated for surgery in the absence of serious concomitant illnesses, despite the tumor dimension. The surgical approach, whenever possible, is the best recognized option to cure the syndromes and to increase the oncologic outcome after optimal medical control of the symptoms [13, 15, 16]. Similarly, bigger NF-PanNETs in fit-for-surgery patients are good candidates for resection. Conversely, there is still an ongoing debate between surgical resection versus observation in the presence of small NF-PanNETs (≤2 cm).

The aim of this paper is to focus on the management of sporadic PanNETs as highlighted by different guidelines and previously published papers.

## 2. Diagnosis and Prognosis of PanNETs

Diagnosis of PanNETs is widely increasing, mostly as incidental, due to the more and more frequent use of high resolution imaging examinations associated with a greater awareness of these pathologies [13, 17]. According to the paper written by Kuo and Salem [1] based on the American Surveillance, Epidemiology and End Results (SEER), the diagnosis of PanNETs smaller than 2 cm has risen from 12% in 1988 to 20% in 2009. A more recent paper on the same database that included 64,971 patients with a NET from 1973 to 2012 showed a global increase in the diagnosis of NETs of sixfold. Nevertheless, within patients with a known tumor grade (70%), 51% had a G1 NET and 16% had a G2 NET. G1 NETs showed the major increase in incidence. Within the patients with a known stage, 52% had a localized disease at diagnosis. This trend was seen across all sites and pancreas as well [17].

The traditional laboratory workup in NF-PanNETs [13] comprehends chromogranin A (CgA), with a sensitivity of 72–100% and a specificity of 50–80%, and neuron-specific enolase (NSE) (sensitivity of 30–40% and a specificity of up to 100%). Their combined evaluation adds strength to their single diagnostic power [18]. However, the routine use of CgA is still questioned for its limited importance in the presence of small lesions. Other tests, such as transcript multianalyte assays, appear as promising and more sensitive and efficient when compared to the single CgA analysis [19, 20]. The appropriate hormone evaluation is to be included if a functional tumor is suspected.

Radiologic imaging comprehends CT (computed tomography) scan or magnetic resonance imaging (MRI), endoscopic ultrasound (EUS) with a fine-needle biopsy [21], and somatostatin receptor-based imaging to localize/stage the neoplasm [13, 16, 22].

Larghi and colleagues [23] performed a prospective study evaluating feasibility and yield of the 19-gauge needle biopsy under EUS guidance. Despite the small sample (30 patients, 10 operated), they found a rate of 83.3% of concordance between preoperative and postoperative Ki-67 indexes.

Mitotic count and Ki-67 expression were the important items to be taken into account in the 2010 WHO classification. Grades 1 and 2 were considered as differentiated tumors (90%, Ki-67< 20%), while Grade 3 were classified as neuroendocrine carcinomas (NEC) [24]. However, more recent evidences [25, 26] demonstrated heterogeneous biology within the G3 subgroup, in which few well-differentiated tumors with Ki-67 > 20% showed a mild prognosis. The updated 2017 WHO classification [27] properly classified these tumors as well-differentiated G3-NETs rather than poorly differentiated G3-NEC [28, 29]. The use of immunohistochemical markers may help in differentiating these two subgroups. This distinction has a therapeutic and prognostic value in such tumors, although their rarity leads to the need of further studies to completely validate this new classification. Moreover, the 2017 WHO classification established the threshold of the Ki-67 index at 3% between G1 and G2 NETs [27]. Furthermore, since the Ki-67 index seems to be not sufficient to classify these tumors, the inclusion of some other genetic mutation analyses is expected in the upcoming classifications [25].

Nevertheless, some different Ki-67 index cut-offs between G1 and G2 have also been proposed (3–10%) [30, 31], and different classification systems have been suggested and revised over the years.

According to a robust comparative study including more than 1000 patients, the American Joint Committee on Cancer (AJCC, 7th edition), the World Health Organization (WHO) 2010, and the European Neuroendocrine Tumor Society (ENETS) classification systems all resulted to be independent prognostic factors for survival, although the ENETS TNM seemed to be the most accurate if compared to the others [32]. On the other hand, Strosberg and coworkers [33] reported the validity of the AJCC system in a study involving 425 patients, reporting a 5-year OS rate of 92%, 84%, 81%, and 57% in case of stages 1 to 4, respectively.

Luo et al. [34, 35] proposed a modified ENETS TNM system using the ENETS TNM definition associated with the AJCC staging definition. Subsequently, their data was validated according to the North American SEER registry, within multicentric series including thousands of patients. However, the AJCC released the 8th edition with the new TNM staging system identical to the ENETS TNM [29].

Several other independent prognostic factors have been recently recognized:

- The presence of calcification at preoperative imaging seems to be related to tumor grade and metastatic lymph node numbers.
- Distant metastases and their progression time are survival predictors, independent from the Ki-67 index [36].
- Lymph node involvement and lymph node ratio are both related to the tumor recurrence after surgery.
- The absence of symptoms in NF-PanNETs seems related with a better prognosis, independent from the tumor stage [13].
- Peritumoral vascular invasion is recently known as an independent prognostic factor [36].
- Older age, with different cut-off (55–75 years), is related with a higher mortality rate [37, 38].

The median and the 5-year overall survival (OS) for patients affected by NF-PanNETs are 38 months and 43%, respectively [39]. The tumor spread is another important prognostic factor, with the median OS falling from 124, 70, and 23 months for patients with localized disease, regional tumor involvement, and metastatic disease, respectively [39].

Interestingly, less than 10% of pancreatic insulinomas are frankly malignant. However, the diameter > 2 cm and Ki-67 > 2% are both predictors of liver metastasis, with the median survival of less than 2 years in this evidence [15]. Furthermore, up to 40% of the patients with gastrinomas develop liver metastasis, representing the most important prognostic factor (10-year OS of 10–20% for metastatic disease and 90–100% for without metastasis) [15].

## 3. Surgery versus Observation of NF-PanNETs

Specific criteria to definitively and unequivocally predict the behaviour of PanNETs have not been found yet. Consequently, the heterogeneous and often unpredictable behaviour of PanNETs leads to a difficult management of these patients.

The most used criteria are size or change in size during the years, morphological aspect, grade, and Ki-67 expression [12, 40]. In brief, the risk of overtreatment (unnecessary pancreatic resection for an indolent neoplasm) should be carefully balanced with the risk of undertreatment (missing the opportunity to cure a mild to more aggressive disease).

Unfortunately, pancreatic surgery still has significant mortality, ranging from 1% to 10% [41], and morbidity, including perioperative and long-term complications (i.e., diabetes, pancreatic exocrine impairment), of up to 50–60%, even in high volume centers [40, 42–46].

Some authors suggested a nonoperative management through a “wait-and-see” policy of “small” NF-PanNETs [2, 36, 47, 48]. The prolonged careful observation of these lesions could avoid pancreatic surgery and its related frequent complications, because most of the small NF-PanNETs are indolent despite a chance of 10% of nodal involvement [47]. Nevertheless, patients with growing tumors during the follow-up may receive subsequent surgery without changes in OS and disease-free survival (DFS) rates [47].

Sadot and colleagues [2] published a matched case-control study of patients with PanNETs smaller than 3 cm who were observed (104 patients) and compared to those who underwent upfront resection (77 patients). Twenty-five per cent of the patients in the observation group underwent subsequent tumor resection after a median interval of 30 months. No patients died for the neoplasm after a median follow-up of 44 months in either group. Interestingly, the authors did not found any difference in OS between the two groups, although the incidence of “salvage surgery” was higher than those reported by other authors. This difference may be related with the chosen bigger cut-off of 3 cm. Nevertheless, in 65% of the cases, indication to surgery was given according to patients' (38%) or physicians' (27%) preferences. They concluded that observation for stable, small, incidentally discovered PanNETs could be reasonable, in selected patients [2].

According to the updated ENET guidelines [13], some patients with NF-PanNETs ≤2 cm could be safely managed conservatively. Additional criteria for the nonoperative approach should be the presence of G1-low G2 tumor, pancreatic head localization, and no signs of malignancy at imaging. In patients with G2 NF-PanNETs of 2 cm, surgery should be recommended. Similarly, patients with tumor bigger than 2 cm should be evaluated for surgery routinely. The presence of concomitant illnesses and patients' age or wishes should be also considered. However, in the case of surveillance, EUS and MRI should be mandatory to be repeated every 6 months (12 months if no changes are discovered). If an increase of 0.5 cm (or more) in the size of the lesion occurs, patients should be reevaluated for surgery [13].

The comparison between observation and upfront surgery in a small case series (35 patients) reported by Rosenberg et al. [49] showed the absence of significant progression in the observed tumors smaller than 2 cm. Unfortunately, the reported median follow-up was only 27.8 months when dealing with mild aggressive tumors. Interestingly, the same authors found no strict relation between Ki-67 index and aggressive behaviour, although many patients had an unknown tumor grade (73% and 5% for observation and resected groups, resp.). However, other authors did not recommend the routine evaluation of Ki-67 in small PanNETs due to its limited value in case of the tumor biopsy [47]. Similarly, the results of a French multicenter study involving 80 patients reported how the tumor size was an independent predictor of malignancy, while the Ki-67 index was not. Again, 18% of the patients had no Ki-67 index evaluation. Furthermore, the authors found that a size cut-off of 1.7 cm had a very high sensitivity and specificity to predict a malignant behaviour (92% and 75%, resp.) [50].

Zhang et al. [51] in a case series of 249 patients (193 resected and 56 observed) reported a significant OS benefit for the resected group. However, the surgical approach became significant predictor of OS for tumors > 1.5 cm only. Analogue size cut-off values were reported in other papers [52].

Conversely, the American National Comprehensive Cancer Network (NCCN) guidelines [16] recommend surgery in every NF-PanNET bigger than 1 cm, and they stated that observation can be considered in incidentally discovered, low-grade NF-PanNETs smaller than 1 cm. Additional factors for conservative management include the surgical risk, the tumor site, and the patient comorbidities, especially when dealing with small asymptomatic tumor [16]. Probably, the more aggressive surgical approach of the Cancer Network professionals could be justified by the target which obtains the best chance of tumor survival for this kind of malignancies.

Similarly, the Canadian National Expert Group suggested a surgical approach for every healthy patients with resectable disease. Surveillance may be considered only in NF-PanNETs smaller than 2 cm, with a low Ki-67 index measured on EUS-FNA samples and no signs of tumor local or distant spread [53].

The rationale for a more aggressive approach (routine surgery) is that some small (<2 cm) high-grade tumors have a frankly malignant behaviour (9% to 39%) [1, 37, 54–58]. Nevertheless, a proper histological examination of the tumor (including mitotic and Ki-67 indexes) is possible only on the resected specimen. Therefore, some authors believe that an upfront surgical treatment, whenever possible (patients fit for surgery), is the best chance of cure, despite the size of the tumor, providing the longer survival [54, 59].

Kuo and Salem [1] reported a population-level analysis of PanNETs <2 cm using the SEER database. They found the presence of some extrapancreatic tumor spread, nodal involvement, or distal metastasis in 17.9%, 27.3%, and 9.1% of the cohort, respectively. The tumor grade (unknown in 47.9%) and patient race were the most significant predictor of DFS. However, the DFS at 5, 10, and 15 years was 89.7%, 80%, and 70.6, respectively.

Gratian and colleagues [54] reported a large population study using the National Cancer Data Base including 1854 patients with NF-PanNETs ≤ 2 cm diagnosed between 1998 and 2011. Tumors ≤ 0.5 cm in their maximum size presented at diagnosis with nodal or distant metastases in 33% and 11% of cases, respectively. Nevertheless, tumor size was positively associated with distant tumor spread. The five-year OS was 27.6% for the observation group versus 83.0%, 72.3%, and 86% (*p* < 0.01) for distal pancreatectomies (DP), pancreaticoduodenectomies (PD), and total pancreatectomies (TP), respectively.

In a recently published review and meta-analysis, Sallinen et al. [41] criticized the low quality of the previously published studies. In this issue, the authors focused the attention on the lack of important data in most of the published articles, including unacceptable low rates of confirmed diagnosis of PanNETs (46% in the studies about sporadic PanNETs). Therefore, definitive conclusions might actually not be drawn. Moreover, the criteria applied in the wait-and-see policy of control arms might include patients' and surgeons' wishes. Nevertheless, the tumor growth was seen in 22% of the patients with sporadic PanNETs (pooled estimate) while none developed metastasis during follow-up period [41]. In the same review, the surgery rate during the follow-up ranged from 3 to 25% with 43% of the patients operated for their or surgeons' preferences rather than for objective parameters. The authors also analysed the huge differences between the results of case series and the studies based on oncological databases. The lack of data regarding tumor-related history and the influence of external factors such as insurance status and the presence of many selection biases led to an underreporting of patients with less aggressive neoplasms. Nevertheless, most of such type databases reported a malignant potential even in small tumors (>0.5 cm) [1, 56, 59, 60]. Lastly, the authors concluded that the brand-new acquisitions on Pan-NETs could lead to a more restrictive indication to surgery [41]. Similar considerations were reported by others [61].

**Figure 1:**
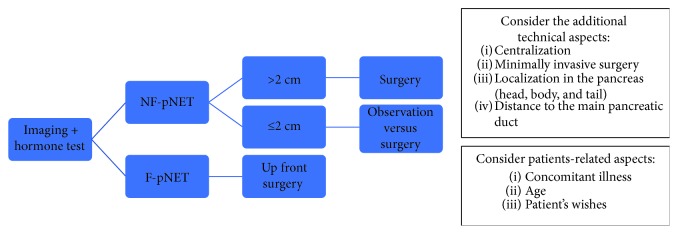
Summary and proposal of a management flow chart in PanNETs.

A proposal of an algorithm is outlined in Figure 1.

## 4. Resective Surgery

### 4.1. Lesion Localization

The preoperative exact localization of the lesion within the pancreatic gland is of crucial importance. According to recent papers, PET (positron emission tomography)/CT with ^68^Ga-labeled somatostatin analogues should be the examination of choice for both staging and localization in noninsulinoma PanNETs and has replaced the suboptimal octreoscan, with a sensitivity and a specificity of 86–100% and 79–100%, respectively [13]. Conversely, sensitivity of PET/CT with ^68^Ga-labeled somatostatin analogues is reported to be around 25% in case of insulinomas [13], reflecting up to 10% of these tumors having a negative preoperative imaging workup. For these patients, selective intra-arterial injection of calcium with hepatic venous insulin gradients has been advocated, although more recent, noninvasive methods of localization have been developed for insulinomas [22]. Several new cellular targets and tracers such as enxendin-4 or ^18^F-FDOPA (6-[18F]-L-fluoro-L-3,4-dihydroxyphenylalanina) have been employed [22].

Benign insulinomas usually express glucagon-like peptide-1 receptors (GLP-1R), and imaging with different radiolabelled-exendin-4 compounds (i.e., ^68^Ga-NOTA-exendin-4) is recommended, with a sensitivity up to 90%. In case of high suspicion of well-differentiated metastatic insulinoma, somatostatin receptor imaging (i.e., ^68^Ga-DOTA-octreotate (^68^Ga-DOTATATE) PET/CT) is also advisable to complete the staging and to assess the feasibility of medical treatment, with a sensitivity of up to 80%. Similarly, ^18^F-FDOPA after premedication with carbidopa may be used, although its role is still controversial [22]. Conversely, FDG-PET (2-[^18^F]fluoro-2-deoxy-D-glucose) is used in the presence of high-grade metastatic insulinomas. Moreover, the shift from GLP-1R to SSTR to FDG avidity is described as a “triple-flop” phenomenon, reflecting a progression from benignity to malignity [22].

The sensitivity of intraoperative ultrasound (IOUS) in the detection of small p-NETs is similar to that of EUS, but if combined with direct palpation, its sensitivity rises to 97% [62].

### 4.2. Parenchymal-Sparing Operations versus Demolitive Operations

**Figure 2:**
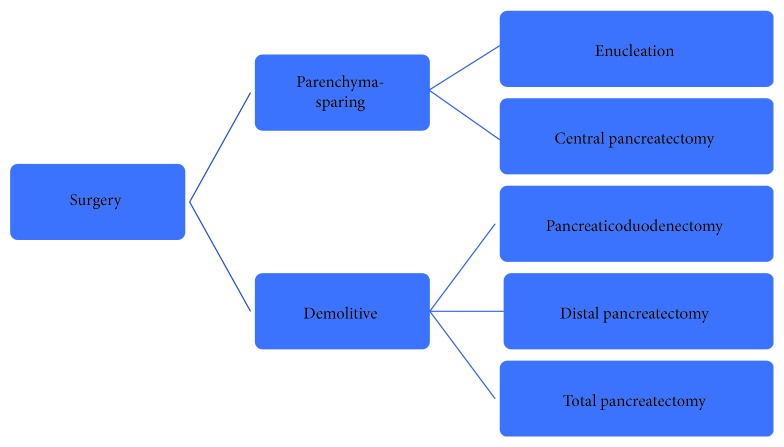
Summary of all surgical options available to deal with PanNETs. A tailored, single-patient, focused approach remains the best option.

There are many different surgical options to deal with PanNETs, ranging from simple enucleation (EN) to a total pancreatectomy [14] (Figure 2).

Obviously, demolitive operations may lead to an unnecessary removal of a huge amount of healthy pancreatic parenchyma and lead to life-threatening postoperative complications, including death.

A rationale strategy for small low-grade malignant tumors could be to remove the tumor only, conserving as much glandular tissue as possible and avoiding lesions of the main pancreatic duct [57, 63–65].

Obviously, the oncological results, including both OS and disease-free survival (DFS), should be equivalent between EN and demolitive surgery, with a proper and detailed surveillance program. Most of the case series and review articles comparing EN and standard surgery reported no differences in the OS and local and distant recurrence rates [66–70]. Some authors reported suboptimal results after EN in terms of increased recurrences in more aggressive tumors located in the head of the pancreas [64]. To achieve these excellent oncologic results, a careful patient selection is required to reserve major pancreatic resection to the more aggressive, large-sized tumors with nodal involvement [63, 67, 71, 72]. The maintenance of the pancreatic endocrine and exocrine functions is the major long-term benefit related to limited surgery (i.e., enucleations) compared to major pancreatic resections (pancreaticoduodenectomy, PD; distal pancreatectomy, DP; and total pancreatectomy, TP) [63, 66, 68, 69, 73–75].

Despite their apparent scarce invasiveness, the major drawback of EN is the high complication rate, mostly related to postoperative pancreatic fistulas (POPFs) [72, 76]. Fortunately, most of them are classified as low grade [63] according to the guidelines of the International Study Group for Pancreatic Fistula (ISGPF) [77] and amenable to be managed conservatively, at the price of prolonged hospital stay and increased costs [72].

The incidence of POPFs is globally reported to be superior after EN with respect to major pancreatic resections, especially if the lesion lied in the head (18%–50% versus 12%) [64, 66–69, 72, 74, 78]. There are many possible explanations to this high rate of POPFs. Firstly, these lesions are often associated with a nondilated pancreatic duct within a soft and friable pancreas. Secondly, the lack of specialization and centralization in high volume hospitals is proven to be related to worst perioperative outcomes. Finally, the localization of the p-NET in the head is a risk factor for POPF after EN, due to the presence of a bigger pancreatic duct.

Interestingly, another concern is represented by tumors arising from the pancreatic head, in which some surgeons could be tempted to push on the technical limits of EN, in order to avoid the challenging PD.

Zhang and coworkers [75] in their case series of 119 patients receiving enucleation (91% for PanNETs) reported that NYHA (New York Heart Association) class II or III and operative time longer than 180 min were both independent risk factors for POPF development.

Compared with major resection, perioperative outcomes of EN were at least equal if not superior, except for a higher rate of POPF. Moreover, minimally invasive EN had a significative shorter operation time and a shorter length of hospital stay if compared to open enucleation [70]. Furthermore, minimally invasive ENs have better results compared to other parenchyma-preserving procedures such as central pancreatectomy, pancreatic head resection, dorsal pancreatectomy, and middle-preserving pancreatectomy [12, 14, 42, 63, 66–69, 71, 73].

Despite the theoretical previous mentioned indications for EN, tumors should also be at least 2-3 mm far from the main pancreatic duct in order to avoid direct injuries and the development of a POPF [63, 70, 75, 76, 79–81]. Preoperative MRCP associated with IOUS and, eventually, intraoperative frozen section examination are all powerful tools to assure the exact location and to confirm the low aggressivity of the lesions [72, 74, 75, 80].

When considering the group of F-PanNET only, EN is considered safe for insulinomas, while gastrinomas were usually candidates to a major pancreatic resection with formal lymphadenectomy due the higher risk of lymph node metastasis (60–90%) and locoregional involvement [16]. Enucleation plus lymphadenectomy, could be considered acceptable only for small exophytic gastrinomas of the pancreatic head, if other preoperative signs of malignancies were excluded [13, 16, 82].

Some authors suggested that EN is a feasible approach in selected (≤2 cm, G1, superficial) NF-PanNETs [53, 65, 75, 83]. Conversely, this approach could lead to a questionable oncological outcome. Indeed, the tumor size seems to be directly related to the probability of lymph node metastasis. Interestingly, NF-PanNETs smaller than 2 cm have a low (7%–26%) but measurable risk of lymph node metastases. In summary, the updated NCCN guidelines and others indicate the cut-off value of 2 cm in diameter to perform pancreatic EN [16, 39]. Significant tumor growth in the previous 3–6 months is another parameter that contraindicates an EN outside specific cases [82].

An impressive meta-analysis collecting 1148 patients (38% of EN and 62% of major resections; minimally invasive technique employed in 25.5% and 22.4%, resp.) with p-NETs or other cystic neoplasms, found that duration of surgery, length of hospital stay, and organ impairment favored EN. Nevertheless, the POPF's rate was significantly higher in the EN group, although morbidity and mortality did not differ [42].

Zhou et al. [70] performed a systematic review including 1316 pancreatic EN for benign or low-grade malignant pancreatic tumors (65.6% of PanNETs) with an overall morbidity of 50.3%, POPF representing the most frequent complication (38.1%). Reoperations were 3.7%; mortality and recurrence were 0.3% and 2.3%, respectively. Endocrine and exocrine insufficiencies were observed in only 2.4% and 1.1% of the patients, respectively. Interestingly, in the studies in which EN was compared to demolitive surgery, an equivalent DFS between the two approaches was found.

### 4.3. Lymphadenectomy

The importance of a formal regional lymphadenectomy is still under debate for PanNETs. Franko et al. [84] published a large population study using the SEER database including 2158 patients with PanNETs diagnosed between 1973 and 2004. Tumor size and nodal status were not found to be predictors of OS. These results are consistent with other earlier papers [38, 54, 85], although it could be related to inadequate lymph node sampling.

More recently, some authors suggested a routinary nodal sampling in PanNETs in order to reduce the possibility of tumor understaging rather than to prolong survival itself [83]. Interestingly, the NCCN guidelines focus on the importance of a correct lymphadenectomy, underlining the possibility of nodal metastasis even in the presence of small (1-2 cm) tumors [16]. Conversely, Yoo and colleagues [86] found that routinary lymphadenectomy may be considered as an overtreatment and not necessary in NET G1.

Other papers reported that node involvement and lymph node ratio are both related to the tumor recurrence after surgery [58, 87–90]. Therefore, a formal lymphadenectomy should be considered in all noninsulinoma F-PanNETs [13], since insulinomas do not require a formal lymphadenectomy for their benignity (up to 90% of the patients) [13]. Nevertheless, Sharpe and colleagues [59] found that lymph nodal positivity (29% of patients who underwent surgery) was associated with a higher mortality rates. In the presence of a suspected gastrinoma, formal regional lymphadenectomy may improve survival reducing the persistence or the spread of the disease [15, 88].

In the presence of NF-PanNETs, tumor size seems to relate with the chance of nodal involvement and, consequently, the need of clearance [16, 39, 91]. Interestingly, an extended lymphadenectomy (beyond or far from the pancreas) was not demonstrated to be of great help, even in the presence of more advanced tumors [92].

### 4.4. Extensive Surgery and Systemic Therapy

The role of splenectomy is another debated issue, although most of the authors agree that it should be avoided if splenic vessels are not involved in the neoplastic tissue [93].

In the presence of advanced or metastatic F-PanNETs, palliative surgery may be indicated to relieve symptoms. To achieve this, the removal of at least 90% of tumor load is advocated. Unresectable liver metastasis could be managed by palliative treatments, including transarterial chemoembolization (TACE), radiofrequency ablation (RFA), or cryoablation [15].

NF-PanNETs with vascular involvement could have a prognostic benefit after demolitive resection in selected patients (up to 62% of 10-year OS rate) with a low morbidity rate [85, 94] if performed in high volume centers.

Distant metastases (mostly in the liver) are detected at the time of first diagnosis in about 30% of the patients and in up to 70% in referral centers due to patient preselection toward more complex situations [16, 95].

In case of liver metastasization, surgery may be indicated in well-differentiated G1-G2 PanNETs [95] and when the primary and metastatic tumors are judged as resectable in one- or two-stage surgery. Accurate evaluation of the volume of the future liver remnant should be performed preoperatively, and the surgical plan should be confirmed with the intraoperative ultrasound evaluation [95]. Of course, a simultaneous PD and a major hepatectomy should be avoided to limit perioperative life-threatening complications.

In the case of a planned two-stage surgery, hepatectomy should be performed as the first step, in order to reduce the risk of perihepatic sepsis [13, 16]. Nevertheless, the presence of suspected additional metastatic sites should be excluded before planning any surgical resection, and the presence of concomitant important comorbidities should be taken into consideration [95]. This very aggressive management (in selected patients) leads to an OS of up to 60–80% with morbidity and mortality rate of 30% and 0–5%, respectively [65, 95, 96]. Surgical debulking with palliative intent may also be considered in very selected patients suffering from NF-PanNETs [16].

Pancreatic G3 NEC are usually indicated for medical treatment (mostly based on cisplatin and etoposide) because of high rate of distant metastasis. Systemic therapy is also indicated in nonresectable disease [95]. Patient's characteristics such as the presence of symptoms, comorbidities, and general conditions together with tumor characteristics (histology, stage, and radiotracer uptake) are the parameters to consider in a multidisciplinary team to make a correct choice of medical treatment.

There are three main different groups of medical therapies available: somatostatin analogues (octreotide, lanreotide), molecularly targeted treatment (everolimus, sunitinib), and chemotherapy with cytostatic/cytotoxic drugs (5-fluorouracil (5-FU), capecitabine, dacarbazine, oxaliplatin, streptozotocin, and temozolomide). Although chemotherapy is pushed afterwards more tolerable and manageable in G1-2 PanNETs, in the case of symptomatic, high burden or G2 rapidly-progressing NETs or NEC, it is still the preferred choice as first-line therapy as the only effective therapy.

There are different commonly used regimens (i.e., temozolomide alone or combined with capecitabine or different combination of 5-FU, doxorubicin, and streptozotocin), although there is not a wide consensus on the best protocol. Most of them are under experimentation in ongoing trials [16].

In the next future, conventional chemotherapy might be tailored on each patient according to the tumor biology, including molecular and genetic patterns.

A recently recognized form of treatment is the peptide receptor radionuclide therapy with labelled somatostatin analogues. Main indications are advanced, inoperable G1 or G2 tumors. Patients with G3 tumors expressing somatostatin receptor may receive this treatment in the presence of the progression of disease or in case of a failure of previous therapies [15, 97].

## 5. Minimally Invasive Surgical Techniques

The well-known advantages of laparoscopy include decrease in postoperative pain, lesser blood loss, lower depression of the immune system leading to faster recovery, and definitively, earlier start of adjuvant therapies if required. Nevertheless, due to its intrinsic complexity, the widespread adoption of such techniques in pancreatic surgery was slower if compared to other subspecialities [98–100].

The robotic technology could overcome some of the technical limitations of pure laparoscopy. The EndoWrist system (instruments articulated with 7 degrees of freedom), motion scaling and tremor filtration, stable and high-definition 3D vision, and ergonomic surgeon position are the main advantages. Some other tools are particularly powerful in pancreatic surgery. An ultrasound flexible integrated probe can be moved by the console surgeon and seen together with operative field in a picture-in-picture mode. The adoption of the near-infrared technology and the fluorescence guidance (Firefly® Technology) is a promising tool for tumor localization, although further evidence is needed to confirm its routinary employment for PanNETs. Intraoperative US together with the fluorescence guidance are both crucial for the localization of the lesions and to define their relation with the surrounding healthy tissue or structures.

All these features partially overcome the absence of a tactile feedback [80]. Further, the last generation of da Vinci Xi® robot (Intuitive Surgical, Sunnyvale, California) has several additional technical advantages as compared to the older systems. However, the major drawbacks and limitations of robotic system are the long operative time and the increased costs. The theoretical reduction of hospital stay and the prompter return to daily activities could balance the economic perspective [12, 51].

Indications for the adoption of the minimally invasive surgery do not obviously differ from those for open or laparoscopic surgery, although may lead to a widening of surgical indications in patients suffering for comorbidities at greater risk of postoperative complications. Moreover, more aggressive PanNETs could be managed safely through a minimally invasive approach, achieving the same oncological results [101].

From a comparative perspective, robotic surgery resulted to be safe, feasible, and at least equal to laparoscopy in pancreatic surgery, resulting in low morbidity and short hospital stay [46, 102–104]. Interestingly, duration of robotic EN is shorter than open EN in most of the published studies [68, 70, 81, 105].

Parenchyma-sparing operations could also be associated with the use of a minimally invasive technique to achieve the less clinical impact for the patients. Conversely, some limited case series reported robotic multivisceral resections for metastatic Pan-NET [106].

Unfortunately, there are very few statistically powered studies comparing open, laparoscopic, and robotic techniques in the area of PanNETs. Most of the experiences are reported in wider case series, often merged with different pancreatic tumors (i.e., cystic lesions) [55, 103, 104]. Moreover, many studies comparing minimally invasive techniques had a mixture of pure laparoscopy and robotics limiting the power of any specific comparison [74, 78, 81, 103, 105, 107]. A robust agreement among surgeons tends to recommend the laparoscopic technique to resect insulinomas [98, 108].

Zhang and colleagues [93] presented their initial experience comparing 43 and 31 patients undergoing robotic or laparoscopic DP for PanNETs. They found a significantly higher rate of spleen preservation (79.1 versus 48.4%, *p* = 0.006), lower risk of excessive blood loss, and greater number of lymph node harvested in the robotic group. All the other perioperative outcomes were comparable.

## 6. Follow-Up

The classical follow-up of patients with PanNETs should include clinical examination, appropriate biochemical markers, and imaging techniques such as CT scan and MRI. Somatostatin receptor-based imaging or PET scan should not be routinary used for surveillance [16].

The scheduling of the exams should be modified according to the tumor grade and stage and tailored in each patient after a multidisciplinary round [39]. Patients with a final histopathological confirmation of localized Pan-NET G1 with R0 surgery could avoid longer follow-up. All the other patients should receive tests once or twice a year for 10 years [16]. Patients with NEC should be reassessed every 3–6 months with advanced imaging.

Unfortunately, most of the patients with an advanced Pan-NET will experience some tumor progression. NCCN guidelines reported a global disease recurrence ranging from 21 to 42% [16]. The Ki-67 index is related to tumor spread, with an increasing risk of progression of 2% for each Ki-67 unit [109].

## 7. Conclusions

The incremental incidental diagnosis of small- to medium-size PanNETs has been leading to many challenging clinical decisions. There is still no unanimity about the optimal management of tumor smaller than 2 cm. Most of these tumors have a good prognosis, although the single behaviour is not always predictable. Specific prognostic criteria are still under examination.

The importance of the Ki-67 index as a prognostic factor to drive any decision-making process is still under debate. The tumor size (with different cut-off values) and the location within the pancreatic gland (head, body, and tail) together with the patient age and wishes and the presence of concomitant illnesses are all parameters to be considered for management. The possibility of under/overtreatment is often possible, leading to any delay in the correct management or to the development of life-threatening complications.

The brand-new available publications and guidelines have, however, made the decision algorithm increasingly easier to understand.

Whenever technically adequate and feasible, the parenchyma-sparing pancreatic resections should be preferred especially in young patients. Pancreatic enucleation is the procedure of choice to avoid perioperative morbidities and to preserve organ function in the long term (endocrine and exocrine). Lymphadenectomy or, at least, lymph nodal sampling seems to be important prognostic factor and should be considered routinely.

Despite its relatively new introduction, most of the pancreatic surgery could be achieved through a minimally invasive approach minimizing postoperative impairment but in the hands of experienced surgeons. The robotic platform is a valuable option in order to overcome the intrinsic limits of traditional laparoscopy.

The role of hospital centralization, the multidisciplinary approach, and the surgeon-related volume of activity are also of crucial impact for the final outcomes.

## References

[1] Kuo E. J., Salem R. R. (2013). Population-level analysis of pancreatic neuroendocrine tumors 2 cm or less in size. *Annals of Surgical Oncology*.

[2] Sadot E., Reidy-Lagunes D. L., Tang L. H. (2016). Observation versus resection for small asymptomatic pancreatic neuroendocrine tumors: a matched case-control study. *Annals of Surgical Oncology*.

[3] Sandvik O. M., Søreide K., Gudlaugsson E., Kvaløy J. T., Søreide J. A. (2016). Epidemiology and classification of gastroenteropancreatic neuroendocrine neoplasms using current coding criteria. *British Journal of Surgery*.

[4] Cheema A., Weber J., Strosberg J. R. (2012). Incidental detection of pancreatic neuroendocrine tumors: an analysis of incidence and outcomes. *Annals of Surgical Oncology*.

[5] Bettini R., Partelli S., Boninsegna L. (2011). Tumor size correlates with malignancy in nonfunctioning pancreatic endocrine tumor. *Surgery*.

[6] Oberg K., Knigge U., Kwekkeboom D., Perren A., on behalf of the ESMO Guidelines Working Group (2012). Neuroendocrine gastro-entero-pancreatic tumors: ESMO Clinical Practice Guidelines for diagnosis, treatment and follow-up. *Annals of Oncology*.

[7] Lawrence B., Gustafsson B. I., Chan A., Svejda B., Kidd M., Modlin I. M. (2011). The epidemiology of gastroenteropancreatic neuroendocrine tumors. *Endocrinology and Metabolism Clinics of North America*.

[8] Yao J. C., Hassan M., Phan A. (2008). One hundred years after “carcinoid”: epidemiology of and prognostic factors for neuroendocrine tumors in 35,825 cases in the United States. *Journal of Clinical Oncology*.

[9] Fraenkel M., Kim M. K., Faggiano A., Valk G. D. (2012). Epidemiology of gastroenteropancreatic neuroendocrine tumours. *Best Practice & Research Clinical Gastroenterology*.

[10] Halfdanarson T. R., Rabe K. G., Rubin J., Petersen G. M. (2008). Pancreatic neuroendocrine tumors (PNETs): incidence, prognosis and recent trend toward improved survival. *Annals of Oncology*.

[11] de Wilde R. F., Edil B. H., Hruban R. H., Maitra A. (2012). Well-differentiated pancreatic neuroendocrine tumors: from genetics to therapy. *Nature Reviews Gastroenterology & Hepatology*.

[12] Liu J. B., Baker M. S. (2016). Surgical management of pancreatic neuroendocrine tumors. *Surgical Clinics of North America*.

[13] Falconi M., Eriksson B., Kaltsas G. (2016). ENETS consensus guidelines update for the management of patients with functional pancreatic neuroendocrine tumors and non-functional pancreatic neuroendocrine tumors. *Neuroendocrinology*.

[14] McKenna L. R., Edil B. H. (2014). Update on pancreatic neuroendocrine tumors. *Gland Surgery*.

[15] Jensen R. T., Cadiot G., Brandi M. L. (2012). ENETS Consensus Guidelines for the management of patients with digestive neuroendocrine neoplasms: functional pancreatic endocrine tumor syndromes. *Neuroendocrinology*.

[16] National Comprehensive Cancer Network Neuroendocrine and adrenal tumor (version 2.2018). https://www.nccn.org/professionals/physician_gls/pdf/neuroendocrine.pdf.

[17] Dasari A., Shen C., Halperin D. (2017). Trends in the incidence, prevalence, and survival outcomes in patients with neuroendocrine tumors in the United States. *JAMA Oncology*.

[18] Lv Y., Han X., Zhang C. (2018). Combined test of serum CgA and NSE improved the power of prognosis prediction of NF-pNETs. *Endocrine Connections*.

[19] Kidd M., Bodei L., Modlin I. M. (2016). Chromogranin A: any relevance in neuroendocrine tumors?. *Current Opinion in Endocrinology, Diabetes, and Obesity*.

[20] Modlin I. M., Drozdov I., Alaimo D. (2014). A multianalyte PCR blood test outperforms single analyte ELISAs (chromogranin A, pancreastatin, neurokinin A) for neuroendocrine tumor detection. *Endocrine-Related Cancer*.

[21] Weynand B., Borbath I., Bernard V. (2014). Pancreatic neuroendocrine tumour grading on endoscopic ultrasound-guided fine needle aspiration: high reproducibility and inter-observer agreement of the Ki-67 labelling index. *Cytopathology*.

[22] Pattison D. A., Hicks R. J. (2017). Molecular imaging in the investigation of hypoglycaemic syndromes and their management. *Endocrine-Related Cancer*.

[23] Larghi A., Capurso G., Carnuccio A. (2012). Ki-67 grading of nonfunctioning pancreatic neuroendocrine tumors on histologic samples obtained by EUS-guided fine-needle tissue acquisition: a prospective study. *Gastrointestinal Endoscopy*.

[24] Klimstra D. S., Armold R., Capella C., Bosman F. T., Carneiro F., Hruban R. H., Theise N. D. (2010). Neuroendocrine neoplasms of the pancreas. *WHO Classification of Tumours of the Digestive System*.

[25] Han X., Xu X., Ma H. (2018). Clinical relevance of different WHO grade 3 pancreatic neuroendocrine neoplasms based on morphology. *Endocrine Connections*.

[26] Milione M., Maisonneuve P., Spada F. (2017). The clinicopathologic heterogeneity of grade 3 gastroenteropancreatic neuroendocrine neoplasms: morphological differentiation and proliferation identify different prognostic categories. *Neuroendocrinology*.

[27] Lloyd R. V., Osamura R. Y., Klöppel G., Rosai J. (2017). *WHO Classification of Tumours of Endocrine Organs*.

[28] Basturk O., Yang Z., Tang L. H. (2015). The high-grade (WHO G3) pancreatic neuroendocrine tumor category is morphologically and biologically heterogenous and includes both well differentiated and poorly differentiated neoplasms. *The American Journal of Surgical Pathology*.

[29] Bergsland E. K., Woltering E. A., Rindi G., Amin M. B., Edge S., Greene F. (2017). Neuroendocrine tumors of the pancreas. American Joint Committee on Cancer 2017. *AJCC Cancer Staging Manual*.

[30] Lowe K., Khithani A., Liu E. (2012). Ki-67 labeling: a more sensitive indicator of malignant phenotype than mitotic count or tumor size?. *Journal of Surgical Oncology*.

[31] Hamilton N. A., Liu T. C., Cavatiao A. (2012). Ki-67 predicts disease recurrence and poor prognosis in pancreatic neuroendocrine neoplasms. *Surgery*.

[32] Rindi G., Falconi M., Klersy C. (2012). TNM staging of neoplasms of the endocrine pancreas: results from a large international cohort study. *Journal of the National Cancer Institute*.

[33] Strosberg J. R., Cheema A., Weber J., Han G., Coppola D., Kvols L. K. (2011). Prognostic validity of a novel American Joint Committee on Cancer Staging Classification for pancreatic neuroendocrine tumors. *Journal of Clinical Oncology*.

[34] Luo G., Javed A., Strosberg J. R. (2017). Modified staging classification for pancreatic neuroendocrine tumors on the basis of the American Joint Committee on Cancer and European Neuroendocrine Tumor Society Systems. *Journal of Clinical Oncology*.

[35] Luo G., Jin K., Cheng H. (2017). Revised nodal stage for pancreatic neuroendocrine tumors. *Pancreatology*.

[36] Landoni L., Marchegiani G., Pollini T. (2017). The evolution of surgical strategies for pancreatic neuroendocrine tumors (Pan-NENs): time-trend and outcome analysis from 587 consecutive resections at a high-volume institution. *Annals of Surgery*.

[37] Cherenfant J., Stocker S. J., Gage M. K. (2013). Predicting aggressive behavior in nonfunctioning pancreatic neuroendocrine tumors. *Surgery*.

[38] Bilimoria K. Y., Talamonti M. S., Tomlinson J. S. (2008). Prognostic score predicting survival after resection of pancreatic neuroendocrine tumors: analysis of 3851 patients. *Annals of Surgery*.

[39] Falconi M., Bartsch D. K., Eriksson B. (2012). ENETS Consensus Guidelines for the management of patients with digestive neuroendocrine neoplasms of the digestive system: well-differentiated pancreatic non-functioning tumors. *Neuroendocrinology*.

[40] Chabot J. (2016). Editorial: pancreatic neuroendocrine tumors: primum non nocere. *Surgery*.

[41] Sallinen V., le Large T. Y. S., Galeev S. (2017). Surveillance strategy for small asymptomatic non-functional pancreatic neuroendocrine tumors – a systematic review and meta-analysis. *HPB: The Official Journal of the International Hepato Pancreato Biliary Association*.

[42] Hüttner F. J., Koessler-Ebs J., Hackert T., Ulrich A., Büchler M. W., Diener M. K. (2015). Meta-analysis of surgical outcome after enucleation *versus* standard resection for pancreatic neoplasms. *British Journal of Surgery*.

[43] Balzano G., Zerbi A., Capretti G., Rocchetti S., Capitanio V., di Carlo V. (2008). Effect of hospital volume on outcome of pancreaticoduodenectomy in Italy. *British Journal of Surgery*.

[44] Gooiker G. A., Lemmens V. E. P. P., Besselink M. G. (2014). Impact of centralization of pancreatic cancer surgery on resection rates and survival. *British Journal of Surgery*.

[45] Hata T., Motoi F., Ishida M. (2016). Effect of hospital volume on surgical outcomes after pancreaticoduodenectomy: a systematic review and meta-analysis. *Annals of Surgery*.

[46] Bencini L., Annecchiarico M., Farsi M. (2015). Minimally invasive surgical approach to pancreatic malignancies. *World Journal of Gastrointestinal Oncology*.

[47] Gaujoux S., Partelli S., Maire F. (2013). Observational study of natural history of small sporadic nonfunctioning pancreatic neuroendocrine tumors. *The Journal of Clinical Endocrinology and Metabolism*.

[48] Lee L. C., Grant C. S., Salomao D. R. (2012). Small, nonfunctioning, asymptomatic pancreatic neuroendocrine tumors (PNETs): role for nonoperative management. *Surgery*.

[49] Rosenberg A. M., Friedmann P., del Rivero J., Libutti S. K., Laird A. M. (2016). Resection versus expectant management of small incidentally discovered nonfunctional pancreatic neuroendocrine tumors. *Surgery*.

[50] Regenet N., Carrere N., Boulanger G. (2016). Is the 2-cm size cutoff relevant for small nonfunctioning pancreatic neuroendocrine tumors: a French multicenter study. *Surgery*.

[51] Zhang I. Y., Zhao J., Fernandez-del Castillo C. (2016). Operative versus nonoperative management of nonfunctioning pancreatic neuroendocrine tumors. *Journal of Gastrointestinal Surgery*.

[52] Kishi Y., Shimada K., Nara S., Esaki M., Hiraoka N., Kosuge T. (2014). Basing treatment strategy for non-functional pancreatic neuroendocrine tumors on tumor size. *Annals of Surgical Oncology*.

[53] Singh S., Dey C., Kennecke H. (2015). Consensus recommendations for the diagnosis and management of pancreatic neuroendocrine tumors: guidelines from a Canadian National Expert Group. *Annals of Surgical Oncology*.

[54] Gratian L., Pura J., Dinan M., Roman S., Reed S., Sosa J. A. (2014). Impact of extent of surgery on survival in patients with small nonfunctional pancreatic neuroendocrine tumors in the United States. *Annals of Surgical Oncology*.

[55] Lombardi M., de Lio N., Funel N. (2015). Prognostic factors for pancreatic neuroendocrine neoplasms (pNET) and the risk of small non-functioning pNET. *Journal of Endocrinological Investigation*.

[56] Haynes A. B., Deshpande V., Ingkakul T. (2011). Implications of incidentally discovered, nonfunctioning pancreatic endocrine tumors: short-term and long-term patient outcomes. *Archives of Surgery*.

[57] Finkelstein P., Sharma R., Picado O. (2017). Pancreatic neuroendocrine tumors (panNETs): analysis of overall survival of nonsurgical management versus surgical resection. *Journal of Gastrointestinal Surgery*.

[58] Ricci C., Casadei R., Taffurelli G. (2013). The role of lymph node ratio in recurrence after curative surgery for pancreatic endocrine tumours. *Pancreatology*.

[59] Sharpe S. M., in H., Winchester D. J., Talamonti M. S., Baker M. S. (2015). Surgical resection provides an overall survival benefit for patients with small pancreatic neuroendocrine tumors. *Journal of Gastrointestinal Surgery*.

[60] Kloppel G. (2011). Classification and pathology of gastroenteropancreatic neuroendocrine neoplasms. *Endocrine-Related Cancer*.

[61] Partelli S., Cirocchi R., Crippa S. (2017). Systematic review of active surveillance versus surgical management of asymptomatic small non-functioning pancreatic neuroendocrine neoplasms. *The British Journal of Surgery*.

[62] Weinstein S., Morgan T., Poder L. (2015). Value of intraoperative sonography in pancreatic surgery. *Journal of Ultrasound in Medicine*.

[63] Beger H. G., Siech M., Poch B., Mayer B., Schoenberg M. H. (2015). Limited surgery for benign tumours of the pancreas: a systematic review. *World Journal of Surgery*.

[64] Jilesen A. P. J., van Eijck C. H. J., Busch O. R. C., van Gulik T. M., Gouma D. J., van Dijkum E. J. M. N. (2016). Postoperative outcomes of enucleation and standard resections in patients with a pancreatic neuroendocrine tumor. *World Journal of Surgery*.

[65] Watzka F. M., Laumen C., Fottner C. (2013). Resection strategies for neuroendocrine pancreatic neoplasms. *Langenbeck's Archives of Surgery*.

[66] Pitt S. C., Pitt H. A., Baker M. S. (2009). Small pancreatic and periampullary neuroendocrine tumors: resect or enucleate?. *Journal of Gastrointestinal Surgery*.

[67] Casadei R., Ricci C., Rega D. (2010). Pancreatic endocrine tumors less than 4 cm in diameter: resect or enucleate? A single-center experience. *Pancreas*.

[68] Cauley C. E., Pitt H. A., Ziegler K. M. (2012). Pancreatic enucleation: improved outcomes compared to resection. *Journal of Gastrointestinal Surgery*.

[69] Hackert T., Hinz U., Fritz S. (2011). Enucleation in pancreatic surgery: indications, technique, and outcome compared to standard pancreatic resections. *Langenbeck's Archives of Surgery*.

[70] Zhou Y., Zhao M., Wu L., Ye F., Si X. (2016). Short- and long-term outcomes after enucleation of pancreatic tumors: an evidence-based assessment. *Pancreatology*.

[71] Sallinen V., Haglund C., Seppänen H. (2015). Outcomes of resected nonfunctional pancreatic neuroendocrine tumors: do size and symptoms matter?. *Surgery*.

[72] Faitot F., Gaujoux S., Barbier L. (2015). Reappraisal of pancreatic enucleations: a single-center experience of 126 procedures. *Surgery*.

[73] Sperti C., Beltrame V., Milanetto A. C., Moro M., Pedrazzoli S. (2010). Parenchyma-sparing pancreatectomies for benign or border-line tumors of the pancreas. *World Journal of Gastrointestinal Oncology*.

[74] Crippa S., Boninsegna L., Partelli S., Falconi M. (2010). Parenchyma-sparing resections for pancreatic neoplasms. *Journal of Hepato-Biliary-Pancreatic Sciences*.

[75] Zhang T., Xu J., Wang T., Liao Q., Dai M., Zhao Y. (2013). Enucleation of pancreatic lesions: indications, outcomes, and risk factors for clinical pancreatic fistula. *Journal of Gastrointestinal Surgery*.

[76] Heeger K., Falconi M., Partelli S. (2014). Increased rate of clinically relevant pancreatic fistula after deep enucleation of small pancreatic tumors. *Langenbeck's Archives of Surgery*.

[77] Bassi C., Marchegiani G., Dervenis C. (2017). The 2016 update of the International Study Group (ISGPS) definition and grading of postoperative pancreatic fistula: 11 years after. *Surgery*.

[78] Song K. B., Kim S. C., Hwang D. W. (2015). Enucleation for benign or low-grade malignant lesions of the pancreas: single-center experience with 65 consecutive patients. *Surgery*.

[79] Brient C., Regenet N., Sulpice L. (2012). Risk factors for postoperative pancreatic fistulization subsequent to enucleation. *Journal of Gastrointestinal Surgery*.

[80] Choi K. S., Chung J. C., Kim H. C. (2014). Feasibility and outcomes of laparoscopic enucleation for pancreatic neoplasms. *Annals of Surgical Treatment and Research*.

[81] Jin J. B., Qin K., Li H. (2016). Robotic enucleation for benign or borderline tumours of the pancreas: a retrospective analysis and comparison from a high-volume centre in Asia. *World Journal of Surgery*.

[82] Ore A. S., Barrows C. E., Solis-Velasco M., Shaker J., Moser A. J. (2017). Robotic enucleation of benign pancreatic tumors. *Journal of Visualized Surgery*.

[83] Falconi M., Zerbi A., Crippa S. (2010). Parenchyma-preserving resections for small nonfunctioning pancreatic endocrine tumors. *Annals of Surgical Oncology*.

[84] Franko J., Feng W., Yip L., Genovese E., Moser A. J. (2010). Non-functional neuroendocrine carcinoma of the pancreas: incidence, tumor biology, and outcomes in 2,158 patients. *Journal of Gastrointestinal Surgery*.

[85] Birnbaum D. J., Turrini O., Vigano L. (2015). Surgical management of advanced pancreatic neuroendocrine tumors: short-term and long-term results from an international multi-institutional study. *Annals of Surgical Oncology*.

[86] Yoo Y. J., Yang S. J., Hwang H. K., Kang C. M., Kim H., Lee W. J. (2015). Overestimated oncologic significance of lymph node metastasis in G1 nonfunctioning neuroendocrine tumor in the left side of the pancreas. *Medicine*.

[87] Lamberti G., Ceccarelli C., Brighi N. (2017). Determination of mammalian target of rapamycin hyperactivation as prognostic factor in well-differentiated neuroendocrine tumors. *Gastroenterology Research and Practice*.

[88] Krampitz G. W., Norton J. A., Poultsides G. A., Visser B. C., Sun L., Jensen R. T. (2012). Lymph nodes and survival in pancreatic neuroendocrine tumors. *Archives of Surgery*.

[89] Zhang X., Lu L., Shang Y. (2017). The number of positive lymph node is a better predictor of survival than the lymph node metastasis status for pancreatic neuroendocrine neoplasms: a retrospective cohort study. *International Journal of Surgery*.

[90] Partelli S., Gaujoux S., Boninsegna L. (2013). Pattern and clinical predictors of lymph node involvement in nonfunctioning pancreatic neuroendocrine tumors (NF-PanNETs). *JAMA Surgery*.

[91] Hashim Y. M., Trinkaus K. M., Linehan D. C. (2014). Regional lymphadenectomy is indicated in the surgical treatment of pancreatic neuroendocrine tumors (PNETs). *Annals of Surgery*.

[92] Conrad C., Kutlu O. C., Dasari A. (2016). Prognostic value of lymph node status and extent of lymphadenectomy in pancreatic neuroendocrine tumors confined to and extending beyond the pancreas. *Journal of Gastrointestinal Surgery*.

[93] Zhang J., Jin J., Chen S. (2017). Minimally invasive distal pancreatectomy for PNETs: laparoscopic or robotic approach?. *Oncotarget*.

[94] Haugvik S. P., Labori K. J., Waage A., Line P. D., Mathisen Ø., Gladhaug I. P. (2013). Pancreatic surgery with vascular reconstruction in patients with locally advanced pancreatic neuroendocrine tumors. *Journal of Gastrointestinal Surgery*.

[95] Pavel M., Baudin E., Couvelard A. (2012). ENETS Consensus Guidelines for the management of patients with liver and other distant metastases from neuroendocrine neoplasms of foregut, midgut, hindgut, and unknown primary. *Neuroendocrinology*.

[96] Keutgen X. M., Nilubol N., Glanville J. (2016). Resection of primary tumor site is associated with prolonged survival in metastatic nonfunctioning pancreatic neuroendocrine tumors. *Surgery*.

[97] Kulke M. H., Anthony L. B., Bushnell D. L. (2010). NANETS treatment guidelines: well-differentiated neuroendocrine tumors of the stomach and pancreas. *Pancreas*.

[98] Al-Kurd A., Chapchay K., Grozinsky-Glasberg S., Mazeh H. (2014). Laparoscopic resection of pancreatic neuroendocrine tumors. *World Journal of Gastroenterology*.

[99] Drymousis P., Raptis D. A., Spalding D. (2014). Laparoscopic versus open pancreas resection for pancreatic neuroendocrine tumours: a systematic review and meta-analysis. *HPB: The Official Journal of the International Hepato Pancreato Biliary Association*.

[100] Haugvik S. P., Marangos I. P., Røsok B. I. (2013). Long-term outcome of laparoscopic surgery for pancreatic neuroendocrine tumors. *World Journal of Surgery*.

[101] Fernandez Ranvier G. G., Shouhed D., Inabnet W. B. (2016). Minimally invasive techniques for resection of pancreatic neuroendocrine tumors. *Surgical Oncology Clinics of North America*.

[102] Milone L., Daskalaki D., Wang X., Giulianotti P. C. (2013). State of the art of robotic pancreatic surgery. *World Journal of Surgery*.

[103] Zureikat A. H., Moser A. J., Boone B. A., Bartlett D. L., Zenati M., Zeh H. J. (2013). 250 robotic pancreatic resections: safety and feasibility. *Annals of Surgery*.

[104] Boggi U., Napoli N., Costa F. (2016). Robotic-assisted pancreatic resections. *World Journal of Surgery*.

[105] Shi Y., Peng C., Shen B. (2016). Pancreatic enucleation using the da Vinci robotic surgical system: a report of 26 cases. *The International Journal of Medical Robotics and Computer Assisted Surgery*.

[106] Calin M. L., Sadiq A., Arevalo G. (2016). The first case report of robotic multivisceral resection for synchronous liver metastasis from pancreatic neuroendocrine tumor: a case report and literature review. *Journal of Laparoendoscopic & Advanced Surgical Techniques*.

[107] Tian F., Hong X. F., Wu W. M. (2016). Propensity score-matched analysis of robotic versus open surgical enucleation for small pancreatic neuroendocrine tumours. *British Journal of Surgery*.

[108] Su A. P., Ke N. W., Zhang Y. (2014). Is laparoscopic approach for pancreatic insulinomas safe? Results of a systematic review and meta-analysis. *Journal of Surgical Research*.

[109] Panzuto F., Boninsegna L., Fazio N. (2011). Metastatic and locally advanced pancreatic endocrine carcinomas: analysis of factors associated with disease progression. *Journal of Clinical Oncology*.

